# CO_2_-elevated cell-free protein synthesis

**DOI:** 10.1016/j.synbio.2022.05.002

**Published:** 2022-05-20

**Authors:** Xiaomei Lin, Caijin Zhou, Ting Wang, Xiaoting Huang, Junxin Chen, Zhixia Li, Jisong Zhang, Yuan Lu

**Affiliations:** a Key Laboratory of Industrial Biocatalysis, Ministry of Education, Department of Chemical Engineering, Tsinghua University, Beijing, 100084, China; b The State Key Lab of Chemical Engineering, Department of Chemical Engineering, Tsinghua University, Beijing, 100084, China

**Keywords:** Cell-free protein synthesis, Carbon dioxide, Tube-in-tube reactor, Metabolomics assay

## Abstract

Gases are the vital nutrition of all organisms as the precursor of metabolism pathways. As a potential biological process, protein synthesis is inevitably regulated by gas transport and utilization. However, the effect of carbon dioxide (CO_2_) present in many metabolic pathways on protein synthesis has not been studied well. In this work, carbon dioxide combined with oxygen was employed for cell-free protein synthesis (CFPS) in the tube-in-tube reactor with precise control of gas concentration. In this *in vitro* system, gases could directly affect the protein synthesis process without transmembrane transport. Varied concentrations of carbon dioxide (0–1%) and constant oxygen concentration (21%) were employed for CFPS to assess the effects. The cell-free reactions with 0.3% CO_2_ and 21% O_2_ showed the highest protein yields. The combined effect of CO_2_ and O_2_ also resulted in relatively high protein expression under high oxygen conditions (0.3% CO_2_ and 100% O_2_). Moreover, metabolomics assays were performed to gain insight into metabolic changes, which showed that CO_2_ slightly improved energy metabolism and redox balance. In particular, the extra supplied CO_2_ activated the decarboxylating reactions and removed toxic metabolites to recover the protein synthesis activity. The exploration of CO_2_ on protein synthesis could provide guiding implications for basic studies and biomanufacturing.

## 1 Introduction

Organisms are capable of sensing changes in ambient levels of gases such as oxygen (O_2_) and carbon dioxide (CO_2_), and cells can make rapid physiological responses to fluctuating levels of gases [1,2]. Of which, the protein synthesis processes were significantly affected by environmental gases. O_2_ is an important gas involved in cell metabolism, and its effect on protein synthesis has been demonstrated [3,4], providing evidence for its effects on protein synthesis.

Another gas CO_2_, also as the necessary metabolites, involves diverse biological processes of cellular anabolism and catabolism. CO_2_ is mainly considered the inescapable product of respiration processes so that always involved in aerobic bioprocesses [2,5,6]. Furthermore, CO_2_ and its hydrated counterpart HCO_3_^−^ serve as substrates for carboxylating or decarboxylating reactions. In addition to interacting in the metabolic reactions, CO_2_ is also the necessary regulator that alters physicochemical properties of proteins, adjusts the internal pH, and even regulates protein production [7,8]. Effects of CO_2_ on cellular growth have already been done in the previous reports [[9], [10], [11]]. However, the existing studies on the effects of CO_2_ are still based on the whole-cell activities. The effects of CO_2_ on biological processes such as protein synthesis are not well known, which still require deeper understanding [8].

The presence of cell membranes makes it impossible to accurately measure the actual intracellular concentration of CO_2_, thus limiting the study of the effects of CO_2_ on cellular activity, especially metabolic changes. In addition to freely diffusing through the cellular membrane, CO_2_ may also accumulate in the cells [12]. For this reason, the extracellular carbon dioxide concentration would not precisely reflect the cellular concentration that is affecting the metabolism. Thus, finding an alternative research method, that can directly reflect the CO_2_ concentration to metabolic pathway without the influence of cellular membrane, is still significant.

Cell-free synthesis systems bypass the hindrance of cell membranes by using cell extracts rather than whole cells. Cell-free synthetic biology, using the catalytic components and holding metabolites for energy generation and protein synthesis from cell extracts, is a common and significant biological tool for studying biological reactions [13]. Without using the intact cells, manipulations can easily be made in these open biological systems [[14], [15], [16], [17]]. The extra supplement can directly affect the biological processes without transmembrane. Coupled with a precise built-in gas controlling reactor, cell-free protein synthesis (CFPS) is a flexible alternative system for the insight investigation of cellular metabolic response to gases [18]. For gas generation and control, a tube-in-tube reactor, consisting of a semipermeable inner tubing and a gas nonpermeable outer tubing, is a powerful tool for the gas-liquid reaction. Due to the high permeability and short mass transfer distance of the inner tube of the tube-in-tube reactor, gases in the outer tube can permeate to the reaction system of the inner tube rapidly in about 30 s [19]. The gas mass flowmeter could control the mass fraction of each gas in the outer tube, so that the cell-free reactions in the inner tube can synthesize proteins in varying concentrations of gas directly. In this protein synthesis platform, gas no longer needs to be transported across the membrane, and no aggregation is formed in the system. In addition, the gas concentration ratio can be regulated more accurately. By bypassing cell membranes and walls, CFPS has significant advantages for precise gas concentration control during biological processes, providing unprecedented freedom for precise physiological analysis [20]. Furthermore, the simple composition of CFPS avoids the interaction between complex intracellular metabolic pathways. The dedicated systems improve protein synthesis efficiency, focus on the gas regulation pathways for the systems and avoid the interference of complex cellular metabolic processes on the target metabolic pathways. Therefore, CFPS is an ideal prototyping system to study the effects of gas concentration on protein synthesis. The combination of CFPS and tube-in-tube reactor allows accurate control of gas concentration and convenient gas measurement during protein synthesis. In this device, the gas control and measurement were convenient, and the cell-free protein synthesis reactions were carried out under precisely controlled gas concentration. In this situation, exploring the effect of gas on protein synthesis was simpler, more accurate, and more conducive to the study of cell metabolism.

To study the effects of CO_2_ on protein synthesis processes, in this work, the open CFPS systems were conducted under precise concentration regulation in various gases conditions using the tube-in-tube reactor. The protein synthesis was measured to study the effects on protein synthesis activity. To gain insight into changes in metabolic pathways and understand the metabolic mechanism of gas affecting protein synthesis, the metabolomic profiles were performed on the presentive samples with different gas treating.

## 2 Materials and methods

### 2.1 Tube-in-tube reactor devices

The tube-in-tube reactor was composed of an inner semipermeable membrane tube and an outer impermeable tube. The inner tube was fabricated by the materials Teflon AF-2400 with an inner diameter of 0.6 mm, and the outer tube was made by polytetrafluoroethylene (PTFE) with an inner diameter of 3.175 mm. Due to the high permeability and short mass transfer distance (0.6 mm) of the inner tube, oxygen could penetrate rapidly into the reaction substrates and saturated the reaction substrates in 30 s. By the syringe pump (Harvard PHD, Holliston, USA), the reaction substrates, including the DNA template and cell extract, were continuously injected into the inner tube of the tube-in-tube reactor (Teflon-AF 2400 inner tube, O.D. 0.8 mm, I.D. 0.6 mm; outer PTFE tube, O.D. 3.175 mm, I.D. 1.59 mm, length 2 m). At the same time, the gases (O_2_, CO_2_, N_2_, Beiwen Gas, Beijing, China) were provided by the compressed gas cylinder and controlled by the gas mass flowmeter (Oushisheng Technology Co., Ltd, Beijing, China). Through the T-type mixer, the gas mixture with a certain volume fraction of CO_2_ and O_2_ entered the outer tube of the reactor. The gas passed the membrane on the surface of the inner tube into the inner tube and participated in the protein synthesis. The protein product flowed out from the outlet of the reactor and immediately entered the online detection equipment to determine fluorescence intensity under different experimental conditions. During the process of protein synthesis, the tube-in-tube reactor was immersed in a water bath to maintain the reaction temperature of 30 °C, and the communication between the pump, mass flow controller, water bath, and online detection equipment for process control and data acquisition were achieved by a LabVIEW computer interface.

### 2.2 Cell extract preparation

For standard cell-free expression reactions, *E. coli* Rosetta (DE3) was fermented in 4 L of 2 × YTP (1.6% tryptone; 1% yeast extraction; 0.5% NaCl; 40 mM K_2_HPO_4_; 22 mM KH_2_PO_4_) containing chloramphenicol (34 μg/mL) at 30 °C, 300 rpm. Cells were harvested in the late logarithmic growth phase (∼4 h, OD600 = 2). Cell pellets were washed with ice-cold S30A buffer (14 mM magnesium glutamate; 60 mM potassium glutamate; 50 mM Tris, pH 7.7) three times. Cells were resuspended in S30A buffer (1 mL buffer for 1 g of wet cells) for disruption in a low-temperature ultra-high pressure homogenizer JN-3000 PLUS, made by Guangzhou Poly Nanobiology Technology Co. After powering on the homogenizer, the hydraulic pump and the main engine were opened, and adjusted the valve to relieve pressure. Then, S30A buffer was added to flush the injection cup, and the pressure regulating valve needed to be adjusted to the required pressure (about 1000 bar), waiting for the buffer to run out. Then the sample could be added for crushing. The high pressure in the equipment ruptured the cell wall and cell membrane of *E. coli*, and its contents could be obtained (noting that the sample should be repeated crushing twice). When the process was done, S30A buffer should be added to clean the sample cup and alcohol should be added to soak the sample cup and pipeline to keep them clean and sterile. Then lysate was centrifuged at 12,000×*g* for 10 min at 4 °C. The supernatant was incubated at 30 °C for 80 min after 3 mM DTT added and centrifuged at 12,000×*g* for 10 min at 4 °C again. Then it was dialyzed in molecular porous membrane tubing (6–8 KD MWCO) for 3 h at 4 °C with magnetic stirring. The dialysate was then centrifuged at 12,000×*g* for 10 min at 4 °C, flash-frozen, and stored at −80 °C [21].

### 2.3 Cell-free reaction

Cell-free reagents for tube-in-tube reactions were assembled on ice as generally described and immediately injected into the inner tube for incubation at 30 °C (Figs. S1–2). The general cell-free reaction mixture consisted of the following components: 30% S30 cell extract (v/v%); 15–20 ng/μL DNA templates; 175 mM potassium glutamate; 10 mM ammonium glutamate; 2.7 mM potassium oxalate monohydrate; 10 mM magnesium glutamate; 50 mM each of 19 amino acids without glutamic acid; 3 mM phosphenol pyruvate (PEP); 1 mM putrescine; 1.5 mM spermidine; 0.33 mM nicotinamide adenine dinucleotide (NAD); 1.2 mM ATP; 0.86 mM each of CTP, GTP and UTP; 0.27 mM coenzyme A; 170 μg/mL tRNA; 34 μg/mL folinic; T7 RNA polymerase prepared from *E. coli* BL21(DE3) cell extract; 2% PEG8000 [21].

### 2.4 Fluorescence measurement and data processing

For fluorescent protein sfGFP measurement, the cell-free samples were diluted and measured in the plate reader (TECAN infinite M200 Pro) with the excitation wavelength at 485 nm and emission at 520 nm.

### 2.5 Metabolomics analysis

The metabolisms of cell-free reactions were analyzed by Metabolomics Facility Center in National Protein Science Technology Center, Tsinghua University. 50 μL of cell-free reactions were added into 2 mL of 80% (v/v) methanol (pre-chilled to − 80 °C) and incubated the plates at − 80 °C for overnight. The mixtures were recentrifuged at 14,000×*g* for 20 min. The supernatant was transferred to a new 1.5-mL tube on dry ice and dried to a pellet at room temperature with Speedvac. Ultimate 3000 UHPLC (Dionex) coupled with Q Exactive (Thermo, CA) was used to perform LC separation. Metabolite analysis was performed on Q Exactive Orbitrap mass spectrometer (Thermo, CA). Identified differential metabolites were uploaded to MetaboAnalyst 5.0 (www.metaboanalyst.ca) for analysis of metabolite pathways (Figs. S3–6) [22].

## 3 Results and discussion

### 3.1 Effects of CO_2_ on the CFPS process and its combined effects with O_2_

**Fig. 1:**
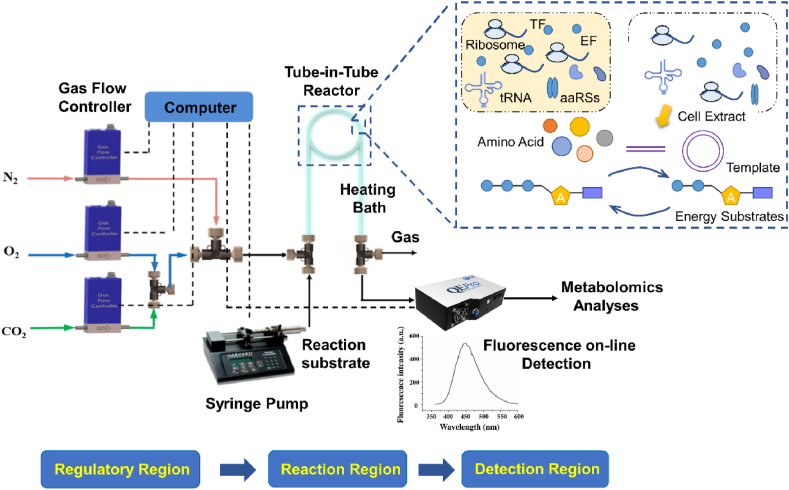
The continuous experimental device diagram based on the tube-in-tube reactor for implementing the flow CFPS process, including three parts: regulatory, reaction, and detection region. TF represented transcription factor, EF represented elongation factor, and aaRSs represented aminoacyl tRNA synthetases.

In the tube-in-tube reactor, the gas can quickly saturate the reaction substrate in the inner tube within 30 s [15], due to its large gas-liquid contact area and shorter mass transfer distance (less than 0.3 mm). In view of the fast gas mass transfer rate, the gas concentration in the reaction substrate can be regarded as constant during the protein synthesis process. In the tube-in-tube reactor, the variation of gas concentration can be easily realized by changing the gas partial pressure in the outer tube. Taking advantage of easy control of the gas concentration in the tube-in-tube reactor, a continuous device including the reactant transport, reaction, and detection regions, as shown in Fig. 1, was built to further study the effects of gas CO_2_ on cell-free protein synthesis. The green fluorescent protein was selected as the reference system.

The gas concentration in the reaction substrate is equal to the oxygen saturation concentration. The gas saturation concentration C* (mg/L) depends on the gas partial pressure in the gas phase and reaction temperature, which is well described by the following Henry's equation [16]:

(1) C*=HP

where *P* (*k*Pa) is the gas partial pressure in the gas phase flowed in the outer tube, and *H* (mg/L·*k*Pa)) is Henry's constant determined by the reaction temperature. When the reaction temperature is constant, the gas partial pressure is easy to change in the tube-in-tube reactor by changing the gas volume flow rate.

**Fig. 2:**
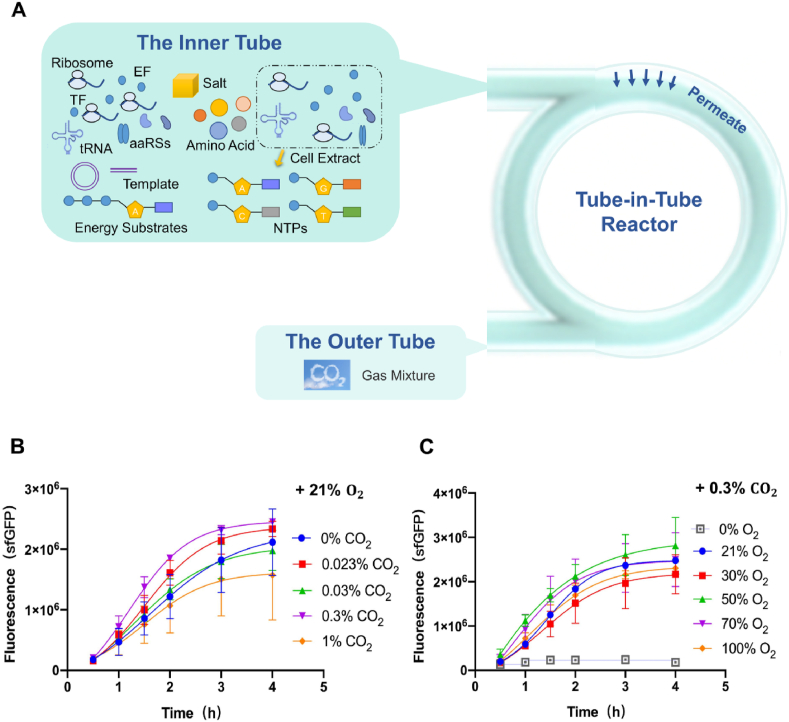
The effects of varying CO_2_ concentrations and their combined effects with O_2_ on CFPS in the tube-in-tube reactor. (A) Schematic diagram of the tube-in-tube reactor for cell-free protein synthesis. TF represented transcription factor, EF represented elongation factor, and aaRSs represented aminoacyl tRNA synthetases. (B) The effects of various CO_2_ concentrations on the CFPS. The oxygen concentration kept consistent: 21% (gas volume fraction). (C) The effects of various O_2_ concentrations on the CFPS. The CO_2_ concentration kept consistent: 0.3% (gas volume fraction). The reaction temperature: 30 °C. The total gas volume flow rate: 100 mL/min.

The protein synthesis platform was constructed by combining an *Escherichia coli*-based CFPS system with the tube-in-tube reactor to measure and analyze the regulation and effects of CO_2_ during protein synthesis. First, the effects of CO_2_ on protein synthesis were studied. Based on our previous work in exposing CFPS systems to an O_2_ gradient of 0–100% [3], the protein expression was the highest with the oxygen concentration of 21%, so oxygen concentration was kept constant at 21%, and CO_2_ concentration was gradually increased from zero. In Fig. 2B, the protein could still be synthesized, when oxygen concentration was 21% without CO_2_. With the addition of CO_2_, it was beneficial to raise protein yield, and the protein concentration was highest when the concentration of CO_2_ increased to 0.3%. Then, the protein product concentration decreased when the CO_2_ concentration was higher than 0.3%. When CO_2_ concentration was increased to 1%, the protein production decreased significantly.

To further study the effects of CO_2_ on protein synthesis, varied concentrations O_2_ were investigated and compared to the previous results. The system was exposed to a 0–100% oxygen gradient at a constant concentration of 0.3% CO_2_, as shown in Fig. 2C. With the addition of CO_2_, the O_2_ concentration of the highest protein expression increased from 21% to 50%, and the protein product concentration also increased with the increase of O_2_ concentration. Noteworthy, although the oxygen concentration was up to 100%, the protein concentration was still high. However, in our previous work, protein concentration was reduced when O_2_ concentration was above 21% with no CO_2_. Especially when O_2_ concentration was 100%, protein expression was almost zero [4]. The different experimental results may be due to the extra effect of CO_2_ on the protein synthesis process. Therefore, a detailed analysis of metabolic processes could be helpful to better understand the combined effects of CO_2_ and O_2_ on protein synthesis.

### 3.2 Metabolomics analyses

**Fig. 3:**
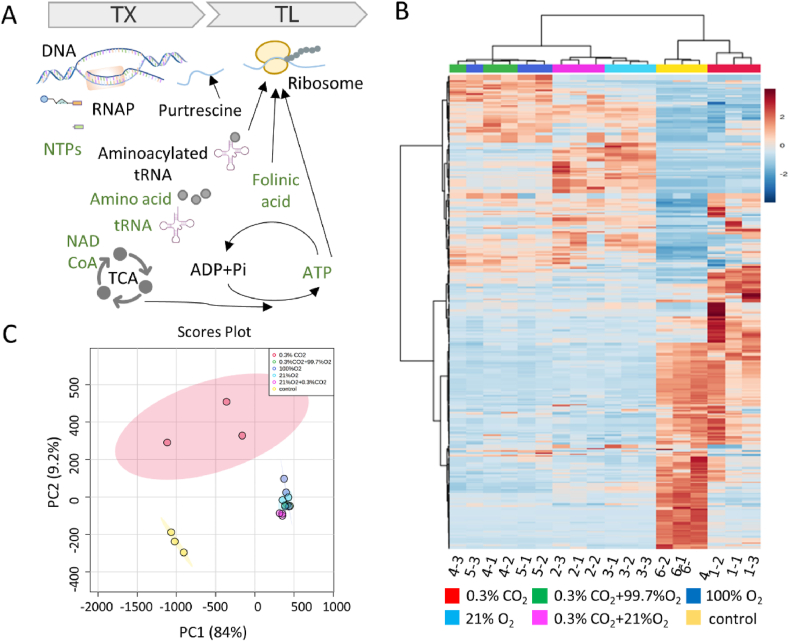
General metabolomics analysis of cell-free reactions with 0.3% CO_2_, 0.3% CO_2_+21% O_2_, 21% O_2_, 0.3% CO_2_+99.7% O_2_, 100% O_2_, and assembled cell-free components. (A) Metabolic system for protein synthesis in cell-free systems. TX represented transcription, and TL represented translation. (B) Heatmap of metabolomics profiles. The first numbers 1–6 in the x-axis respectively represented the above six gas conditions, and the second numbers 1–3 represented the three parallel samples. The columns in the heat map were arranged according to the clustering of the samples, and similar samples were arranged together. The cluster tree was at the top of the heat map. (C) PCA analysis to visualize group discrimination in a 2-dimensional scores plot showed a separation between 0.3% CO_2_ and the control group (assembled cell-free components). Data were collected from at least three replicates.

To study the insight effects of CO_2_ on CFPS, metabolomics assays were employed. In the assembled cell-free reactions, extra supplies were added for the transcription-translation processes, including DNA template, RNA polymerase, NTPs, amino acid, salts, and other substances contained in cell extracts such as purtrescine, ribosome, tRNA, aminoacylated tRNA, NAD, CoA, folinic acid, energy substance ATP, and so on (Fig. 3A). The extra additives as well as endogenous metabolites changed in varying reaction conditions. To explore the changes of the crucial metabolites for protein synthesis, metabolomics assays were performed on cell-free reactions under varied gas concentration conditions for 4 h.

According to the performance of cell-free reactions, it seemed that although the protein synthesis in 0.3% CO_2_ was still weak, there was a synthesis improvement in 21% O_2_ plus 0.3% CO_2_ and high protein concentration in 100% O_2_ plus 0.3% CO_2_. The metabolisms of cell-free reactions with 0.3% CO_2_, 21% O_2_, 0.3% CO_2_+21% O_2_, 100% O_2,_ and 99.7% O_2_+0.3% CO_2_ were performed to study the insight effects of CO_2_ and O_2_. In addition to analysis of completed cell-free reactions, the metabolomics assays were also performed on the assembled cell-free components without protein expression to serve as a control.

A total 568 metabolites were detected in cell-free reactions (Dataset S1). The heatmap of samples obtained from metabolic analysis discriminated the radio of CO_2_, and O_2_ indeed affected the metabolite abundance of cell-free reactions (Fig. 3B). The samples treated with the O_2_ were intersected together in principal component analysis (PCA), an unsupervised dimensional reduction approach that identified the modes of the data (Fig. 3C). In the process of cell-free expression under different gas conditions, metabolites varied with the increase of reaction time. For example, many metabolites (3-hydroxy-3-methylglutarate, levoglucosan, cytidine monophosphate N-acetylneuraminic acid, acetylphosphate, and so on) were lost in the incubated cell-free reactions compared to assembled cell-free components.

**Fig. 4:**
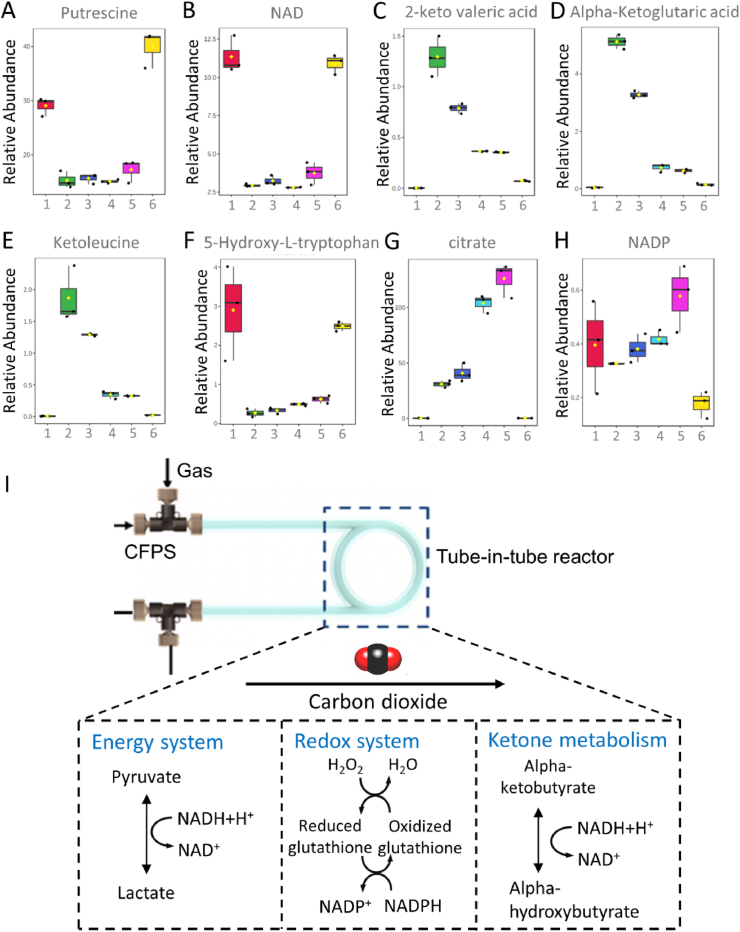
Levels of individual metabolites in completed cell-free reactions and assembled cell-free components. Relative abundance of (A) putrescine, (B) NAD, (C) 2-keto valeric acid, (D) alpha-ketoglutaric acid, (E) ketoleucine, (F) 5-Hydroxy-l-tryptophan, (G) citrate, and (H) NADP. Numbers 1–6 in x-axis were represent sample incubated in gases of 0.3% CO_2_, 21% O_2_, 0.3% CO_2_+21% O_2_, 100% O_2_, 0.3% CO_2_+99.7% O_2_, and assemble cell-free components, respectively. Black lines were the medians, boxes were the middle 50% values, black dots were individual sample levels, and yellow diamonds were the mean. Error bars represented the standard deviation of biological triplicates. (I) The effects of CO_2_ in cell-free metabolic processes.

Moreover, purtrescine and NAD were more concentrated in the samples treated with 0.3% CO_2_ even rather than the assembled group, which were the extra addition in our assembled cell-free reaction for the functional protein synthesis (Fig. 4A and B). To study the mechanism by which the addition of CO_2_ affected the protein expression at the metabolic level, the metabolomics analysis was performed between samples between 21% O_2_ and 21% O_2_+0.3% CO_2_. Seven individual metabolites (2-keto valeric acid, alphe-ketoglutaric acid, ketoleucine, carboxylases, 3-dehydroshikimate, 3-methyl-2-oxobutrate, acide oxoglutanic acid, and glutarate semialdehyde) selected from the metabolites of the above two samples were detected in higher concentration in 21% O_2_ samples without CO_2_. Three of these metabolites were classified to ketone (2-keto valeric acid, alphe-ketoglutaric acid, and ketoleucine). The 2-keto valeric acid and ketoleucine were considered as toxic organic compounds generated as intermediate metabolites. Alphe-ketoglutaric acid played an important role in the metabolic process as an intermediate in the tricarboxylic acid cycle. It was not only a precursor for the synthesis of various amino acids and proteins, but also released a large amount of energy to participate in the biological oxidation through dehydrogenation to produce NADH. However, the three ketone compounds were in lower concentrations in samples treated with 0.3% CO_2_ (Fig. 4C–E). The other 3-dehydroshikimate, 3-methyl-2-oxobutrate, acide oxoglutanic acid, and glutarate semialdehyde were classified to carboxylic acid, which could be catalyzed by carboxylases. Carboxylases, activated by carbon dioxide, were reported to be capable to catalyze ketone to other compound (such as alkenes, alkanes, and secondary alcohols) by carboxylation reactions [12], and it could also catalyze carboxylic acid species. Therefore, the reduction of metabolites in cell-free systems containing CO_2_ might be the result of the activation of carboxylases by CO_2_.

The expression of cell-free reactions treated with 99.7% O_2_ and 0.3% CO_2_ could produce protein, which was different from the previous work with nearly no transcription in high oxygen concentration (100%). In this case, the metabolite analysis was performed between the samples treated with 100% O_2_ and 99.7% O_2_ coupled with 0.3% CO_2_. Among the samples, a few metabolites (NAD, 5-Hydroxy-L-trypophan, citrate, and NADP) were more concentrated in the 99.7% O_2_ and 0.3% CO_2_ rather than that in 100% O_2_ (Fig. 4B and F). As the incubation going, the additional NADH in the cell-free reactions was consumed for the protein expression. However, the high concentration of O_2_ might accumulate the degradation of NADH instead of supplying the reducing agent for protein expression. The CO_2_ could reduce the degradation of NADH, which could maintain the protein expression in high O_2_ samples. The high concentration of other metabolites in CO_2_-containing cell-free systems under high O_2_ conditions may also result from the reduction degradation, thus maintaining protein expression under high O_2_ conditions.

## 4 Conclusion

Although CO_2_ was considered a metabolite, the role of CO_2_ in life has been mainly studied in the whole cell, and its effects on specific metabolic processes such as protein synthesis have not been studied. The presence of cell membranes makes it difficult to accurately measure intracellular concentrations of gases. To meet the above challenges, an open CFPS system was used to synthesize proteins in a tube-in-tube reactor under different gas conditions. Without intact cells, the gas concentration in cell-free reactions can be accurately measured, and the gas mass flowmeter can accurately control the input gas concentration.

In this work, CFPS was conducted in varying concentrations of CO_2_ (0%, 0.023%, 0.03%, and 1% CO_2_) with 21% O_2_. The results showed that, in the gas containing 0.023%, especially 0.3% CO_2_, more proteins were produced, but the protein production decreased with further CO_2_ increase. Moreover, the effect of CO_2_ on protein synthesis with varied concentration of O_2_ was also studied. The addition of CO_2_ to the high O_2_ treatment (99.7% O_2_ and 0.3% CO_2_) resulted in the recovery of protein production compared to 100% pure oxygen.

Metabolomics analysis of gas-treated cell-free reactions showed that metabolites involved in energy generation and redox equilibrium. NAD, NADP and citric acid were more abundant in the 0.3% CO_2_-treated groups. CO_2_-activated decarboxylation caused ketones to exhibit lower concentrations in reactions treated with 0.3% CO_2_, such as 2-keto valeric acid, alpy-ketoglutaric acid, and ketoleucine. In addition, the addition of CO_2_ promoted cell-free protein synthesis under high O_2_ conditions. Appropriately increased CO_2_ activated the removal of toxic intermediates and balanced the redox environment during O_2_ treatment, resulting in higher protein expression (Fig. 4I).

It could be seen that CO_2_ played an important role in metabolic regulation. The adjustment of O_2_ concentration greatly raised the cell-free reactions in reactors, and the additional CO_2_ further elevated the protein synthesis. CFPS systems have been developed as important prototyping tools and biomanufacturing platforms. The combination of cell-free system and tube-in-tube reactor had built a platform with excellent gas transmission performance and open biological systems, in which accurate gas regulations on biological processes could be conveniently investigated. The combined platform not only helps us understand the basic biological gas-utilizing processes, but also improves the CFPS yield for better biomanufacturing purposes. In particular, the platform has the potential to achieve basic understanding and clinical applications through different gas regulations in biological processes.

## Ethics approval

This article does not contain any studies with human participants or experimental animals performed by any of the authors.

## CRediT authorship contribution statement

**Xiaomei Lin:** Investigation, Writing – original draft, preparation. **Caijin Zhou:** Investigation, Writing – original draft, preparation. **Ting Wang:** Investigation, Writing – original draft, preparation. **Xiaoting Huang:** Data curation. **Junxin Chen:** Data curation. **Zhixia Li:** Data curation. **Jisong Zhang:** Supervision. **Yuan Lu:** Writing – review & editing, Supervision, Project administration, Funding acquisition.

## Declaration of interests

The authors declare that they have no known competing financial interests or personal relationships that could have appeared to influence the work reported in this paper.
