# Supplementary material for: CO2-elevated cell-free protein synthesis
Source: Synth Syst Biotechnol. 2022 May 20;7(3):911–7. doi: 10.1016/j.synbio.2022.05.002 (PMC9136254; doi:10.1016/j.synbio.2022.05.002)
Supplement: Multimedia component 1 [file mmc1.docx]

**Supporting Information**

**CO_2_-elevated cell-free protein synthesis**

Xiaomei Lin^1,#^, Caijin Zhou^2,#^, Ting Wang^1,#^, Xiaoting Huang^2^, Junxin Chen^2^, Zhixia Li^1^, Jisong Zhang^2,*^, Yuan Lu^1,*^

^1^Key Laboratory of Industrial Biocatalysis, Ministry of Education, Department of Chemical Engineering, Tsinghua University, Beijing 100084, China.

^2^The State Key Lab of Chemical Engineering, Department of Chemical Engineering, Tsinghua University, Beijing 100084, China.

# These authors contributed equally to this work.

*** Correspondence:** jiszhang@tsinghua.edu.cn (Jisong Zhang); yuanlu@tsinghua.edu.cn (Yuan Lu).

**Supplementary Plasmid Information**

pET23a-sfGFP-6xHis:

tggcgaatgggacgcgccctgtagcggcgcattaagcgcggcgggtgtggtggttacgcgcagcgtgaccgctacacttgccagcgccctagcgcccgctcctttcgctttcttcccttcctttctcgccacgttcgccggctttccccgtcaagctctaaatcgggggctccctttagggttccgatttagtgctttacggcacctcgaccccaaaaaacttgattagggtgatggttcacgtagtgggccatcgccctgatagacggtttttcgccctttgacgttggagtccacgttctttaatagtggactcttgttccaaactggaacaacactcaaccctatctcggtctattcttttgatttataagggattttgccgatttcggcctattggttaaaaaatgagctgatttaacaaaaatttaacgcgaattttaacaaaatattaacgtttacaatttcaggtggcacttttcggggaaatgtgcgcggaacccctatttgtttatttttctaaatacattcaaatatgtatccgctcatgagacaataaccctgataaatgcttcaataatattgaaaaaggaagagtatgagtattcaacatttccgtgtcgcccttattcccttttttgcggcattttgccttcctgtttttgctcacccagaaacgctggtgaaagtaaaagatgctgaagatcagttgggtgcacgagtgggttacatcgaactggatctcaacagcggtaagatccttgagagttttcgccccgaagaacgttttccaatgatgagcacttttaaagttctgctatgtggcgcggtattatcccgtattgacgccgggcaagagcaactcggtcgccgcatacactattctcagaatgacttggttgagtactcaccagtcacagaaaagcatcttacggatggcatgacagtaagagaattatgcagtgctgccataaccatgagtgataacactgcggccaacttacttctgacaacgatcggaggaccgaaggagctaaccgcttttttgcacaacatgggggatcatgtaactcgccttgatcgttgggaaccggagctgaatgaagccataccaaacgacgagcgtgacaccacgatgcctgcagcaatggcaacaacgttgcgcaaactattaactggcgaactacttactctagcttcccggcaacaattaatagactggatggaggcggataaagttgcaggaccacttctgcgctcggcccttccggctggctggtttattgctgataaatctggagccggtgagcgtgggtctcgcggtatcattgcagcactggggccagatggtaagccctcccgtatcgtagttatctacacgacggggagtcaggcaactatggatgaacgaaatagacagatcgctgagataggtgcctcactgattaagcattggtaactgtcagaccaagtttactcatatatactttagattgatttaaaacttcatttttaatttaaaaggatctaggtgaagatcctttttgataatctcatgaccaaaatcccttaacgtgagttttcgttccactgagcgtcagaccccgtagaaaagatcaaaggatcttcttgagatcctttttttctgcgcgtaatctgctgcttgcaaacaaaaaaaccaccgctaccagcggtggtttgtttgccggatcaagagctaccaactctttttccgaaggtaactggcttcagcagagcgcagataccaaatactgtccttctagtgtagccgtagttaggccaccacttcaagaactctgtagcaccgcctacatacctcgctctgctaatcctgttaccagtggctgctgccagtggcgataagtcgtgtcttaccgggttggactcaagacgatagttaccggataaggcgcagcggtcgggctgaacggggggttcgtgcacacagcccagcttggagcgaacgacctacaccgaactgagatacctacagcgtgagctatgagaaagcgccacgcttcccgaagggagaaaggcggacaggtatccggtaagcggcagggtcggaacaggagagcgcacgagggagcttccagggggaaacgcctggtatctttatagtcctgtcgggtttcgccacctctgacttgagcgtcgatttttgtgatgctcgtcaggggggcggagcctatggaaaaacgccagcaacgcggcctttttacggttcctggccttttgctggccttttgctcacatgttctttcctgcgttatcccctgattctgtggataaccgtattaccgcctttgagtgagctgataccgctcgccgcagccgaacgaccgagcgcagcgagtcagtgagcgaggaagcggaagagcgcctgatgcggtattttctccttacgcatctgtgcggtatttcacaccgcatatatggtgcactctcagtacaatctgctctgatgccgcatagttaagccagtatacactccgctatcgctacgtgactgggtcatggctgcgccccgacacccgccaacacccgctgacgcgccctgacgggcttgtctgctcccggcatccgcttacagacaagctgtgaccgtctccgggagctgcatgtgtcagaggttttcaccgtcatcaccgaaacgcgcgaggcagctgcggtaaagctcatcagcgtggtcgtgaagcgattcacagatgtctgcctgttcatccgcgtccagctcgttgagtttctccagaagcgttaatgtctggcttctgataaagcgggccatgttaagggcggttttttcctgtttggtcactgatgcctccgtgtaagggggatttctgttcatgggggtaatgataccgatgaaacgagagaggatgctcacgatacgggttactgatgatgaacatgcccggttactggaacgttgtgagggtaaacaactggcggtatggatgcggcgggaccagagaaaaatcactcagggtcaatgccagcgcttcgttaatacagatgtaggtgttccacagggtagccagcagcatcctgcgatgcagatccggaacataatggtgcagggcgctgacttccgcgtttccagactttacgaaacacggaaaccgaagaccattcatgttgttgctcaggtcgcagacgttttgcagcagcagtcgcttcacgttcgctcgcgtatcggtgattcattctgctaaccagtaaggcaaccccgccagcctagccgggtcctcaacgacaggagcacgatcatgcgcacccgtggccaggacccaacgctgcccgagatctcgatcccgcgaaattaatacgactcactatagggagaccacaacggtttccctctagaaataattttgtttaactttaagaaggagatatacatATGCGTAAAGGCGAAGAGCTGTTCACTGGTGTCGTCCCTATTCTGGTGGAACTGGATGGTGATGTCAACGGTCATAAGTTTTCCGTGCGTGGCGAGGGTGAAGGTGACGCAACTAATGGTAAACTGACGCTGAAGTTCATCTGTACTACTGGTAAACTGCCGGTACCTTGGCCGACTCTGGTAACGACGCTGACTTATGGTGTTCAGTGCTTTGCTCGTTATCCGGACCATATGAAGCAGCATGACTTCTTCAAGTCCGCCATGCCGGAAGGCTATGTGCAGGAACGCACGATTTCCTTTAAGGATGACGGCACGTACAAAACGCGTGCGGAAGTGAAATTTGAAGGCGATACCCTGGTAAACCGCATTGAGCTGAAAGGCATTGACTTTAAAGAAGACGGCAATATCCTGGGCCATAAGCTGGAATACAATTTTAACAGCCACAATGTTTACATCACCGCCGATAAACAAAAAAATGGCATTAAAGCGAATTTTAAAATTCGCCACAACGTGGAGGATGGCAGCGTGCAGCTGGCTGATCACTACCAGCAAAACACTCCAATCGGTGATGGTCCTGTTCTGCTGCCAGACAATCACTATCTGAGCACGCAAAGCGTTCTGTCTAAAGATCCGAACGAGAAACGCGATCATATGGTTCTGCTGGAGTTCGTAACCGCAGCGGGCATCACGCATGGTATGGATGAACTGTACAAACATCACCATCACCATCATTAAgtcgacaagcttgcggccgcactcgagcaccaccaccaccaccactgagatccggctgctaacaaagcccgaaaggaagctgagttggctgctgccaccgctgagcaataactagcataaccccttggggcctctaaacgggtcttgaggggttttttgctgaaaggaggaactatatccggat

f1 origin

AmpR promotor

Ampicillin resistance marker

sfGFP

6xHis

**Supplementary Data**

Specific data were shown in Dataset S1. In the untargeted metabolomics detection data of the high-resolution mass spectrometer, the sample data with a peak area of more than 5$\times{10}^{6}$ was more accurate quantitatively. The secondary matched samples had a secondary Library Score (%). The higher the score was, the better the matching degree was, and the more reliable the data was. Based on the above evaluation criteria for data accuracy, we finally screened the peak area data of 254 metabolites in Dataset S1 for subsequent analysis. The value under each sample was the corresponding peak area of the metabolite in first-level data.

**Supplementary Figures**


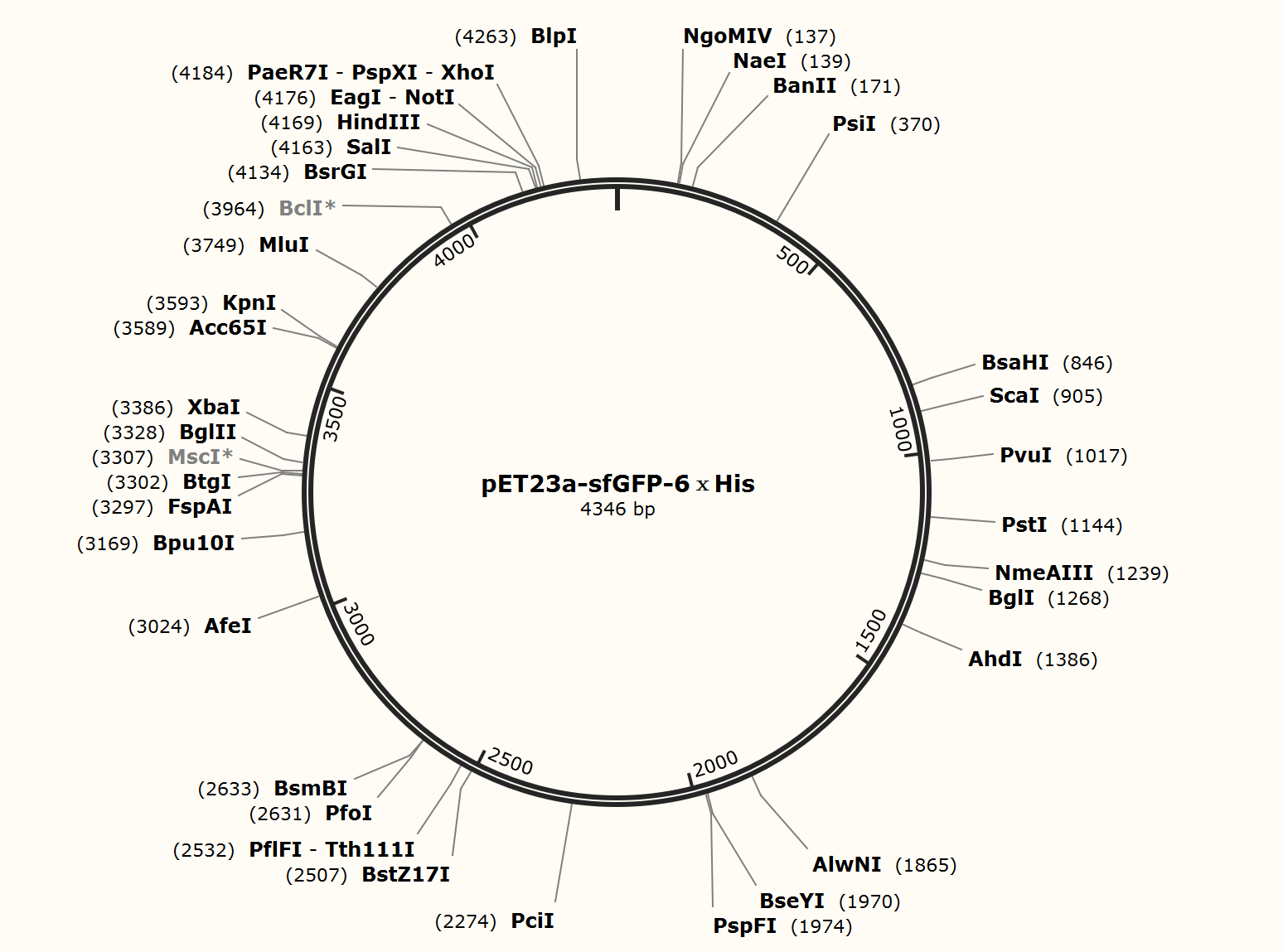


**Fig. S1** The sfGFP (superfolded green fluorescent protein) plasmid map. sfGFP was regulated by a phage-derived T7 promoter and terminator in the backbone of pET23a.

**
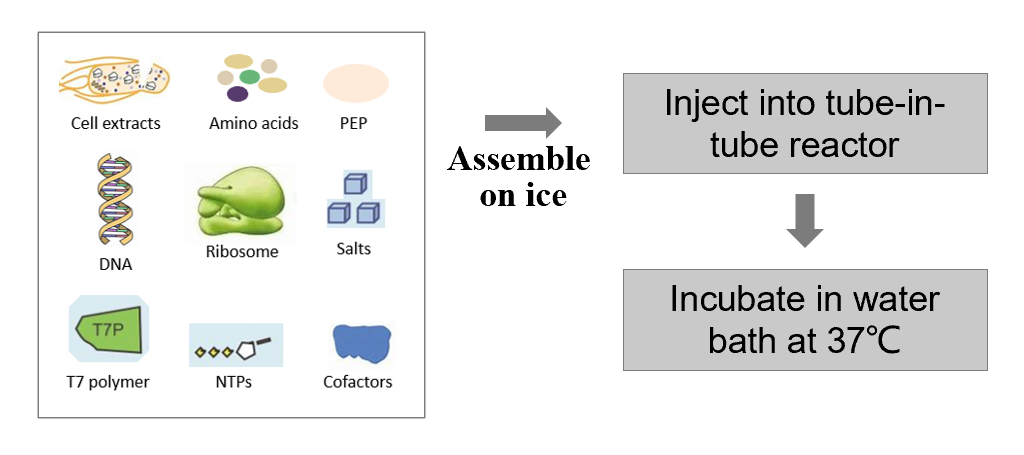
**

**Fig. S2** Workflow for cell-free reactions. Cell-free reaction components (cell extracts, 19 amino acids, PEP, ribosome, salts, T7 RNA polymerase, NTPs, cofactors, and DNA template) were assembled as cell-free protein expression systems on the ice. The systems were injected into the tube-in-tube reactor for protein synthesis at 37 ℃ in a water bath.

**
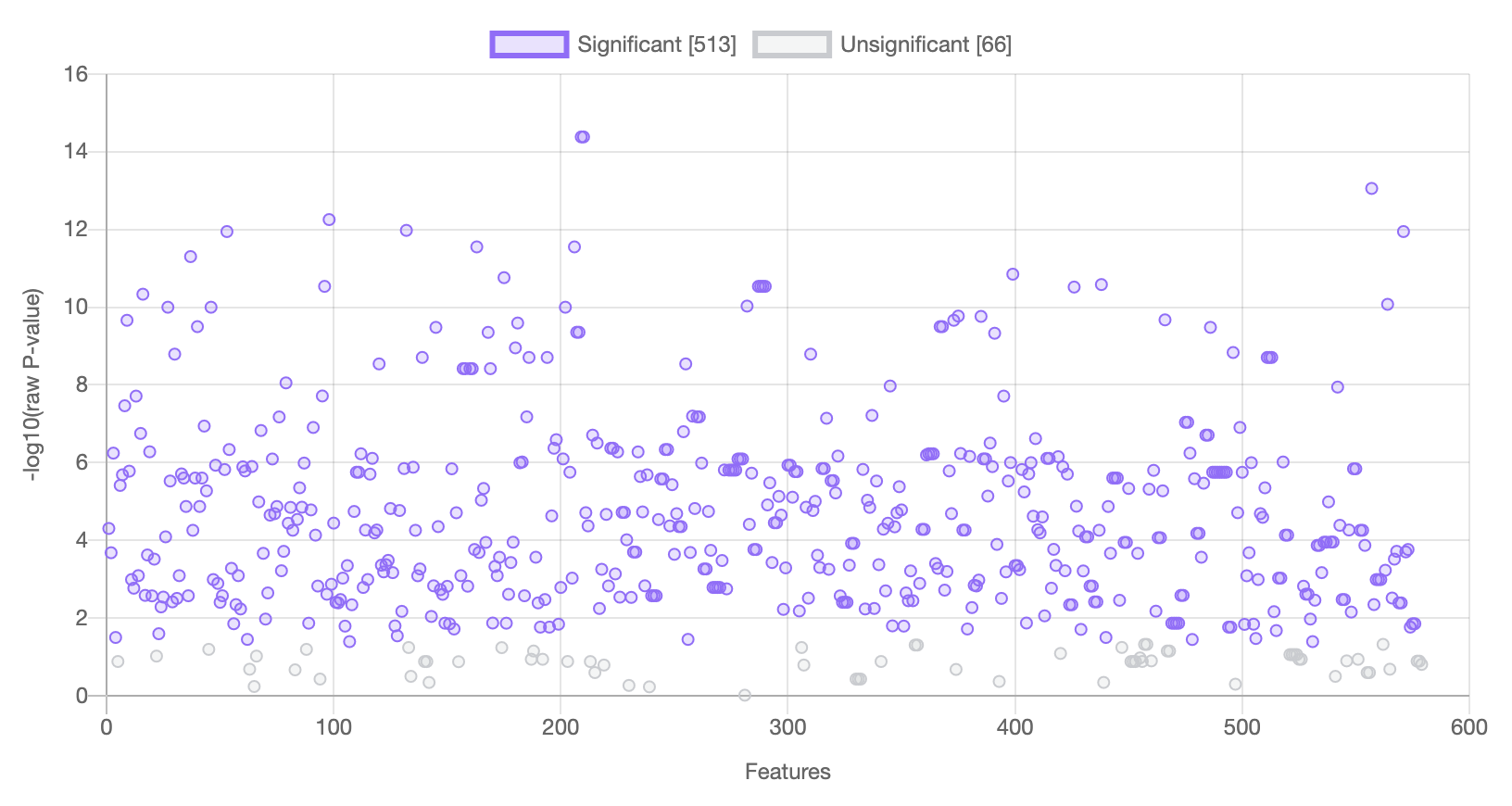
**

**Fig. S3** The significant differential metabolites in cell-free reactions. MetaboAnalyst was used for statistical and pathway analysis. After all data processing, these metabolomics measurements yielded relative abundances for 513 identified and unannotated analytes that were used for further analysis.


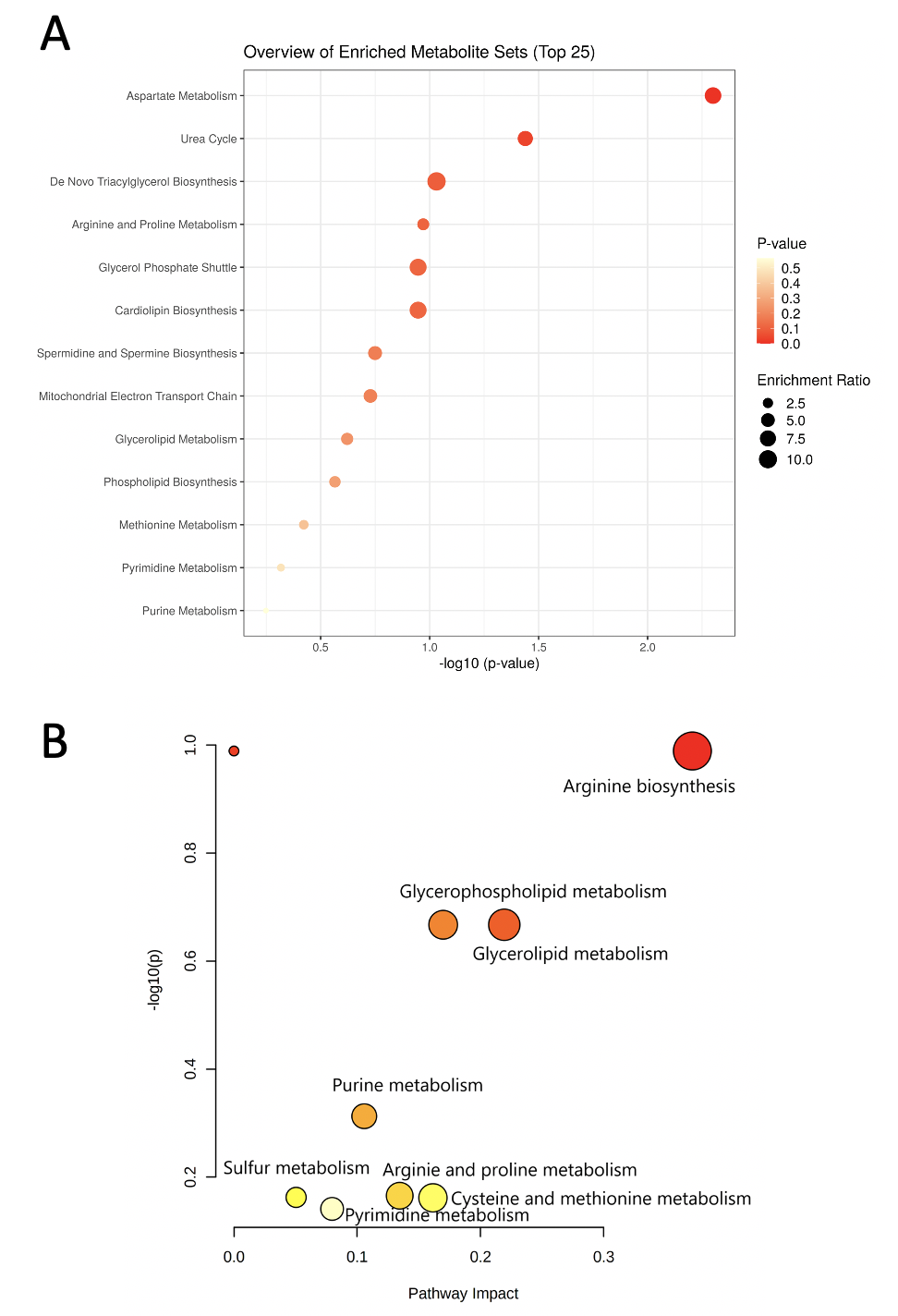


**Fig. S4** Pathway enrichment of down-regulated metabolites between 21% O_2_+0.3%CO_2_ and 21%O_2_. (A) The overview of enriched metabolite set. (B) Impact of enriched pathways. The Y-axis was based on the P-value (derived from pathway enrichment analysis), and the X-axis was based on the pathway impact value (derived from pathway topology analysis). Node color was based on P-value, from light to dark; P-value was from large to small; node radius was based on its pathway impact value, from small to large; impact value was from small to large. The P-values of pathway enrichment analysis and pathway impact values of pathway topology analysis showed all the matched pathways.


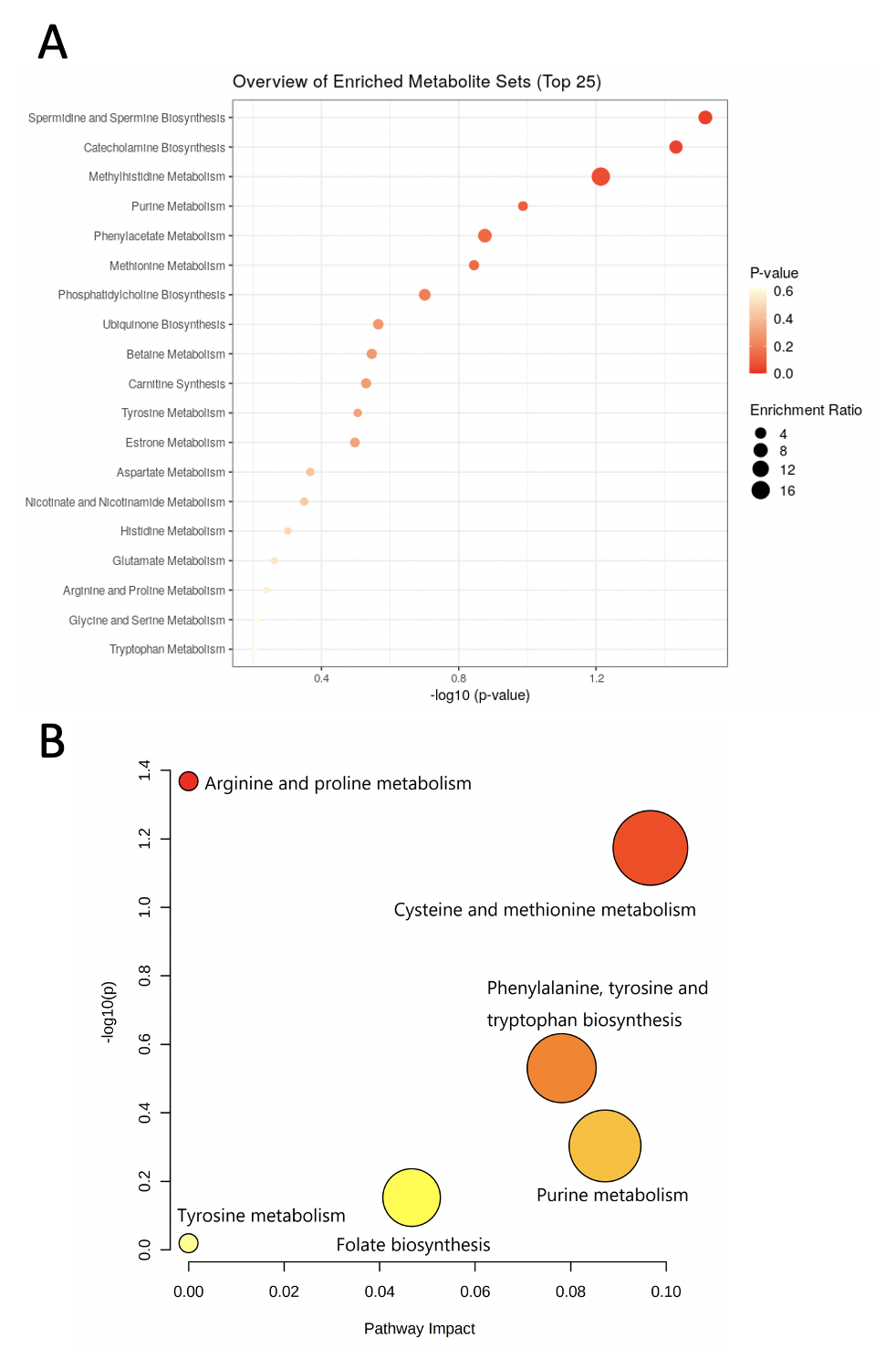


**Fig. S5** Pathway enrichment of up-regulated metabolites between 21% O_2_+0.3%CO_2_ and 21%O_2_. (A) The overview of enriched metabolite set. (B) Impact of enriched pathways. The Y-axis was based on the P-value (derived from pathway enrichment analysis), and the X-axis was based on the pathway impact value (derived from pathway topology analysis). Node color was based on P-value, from light to dark; P-value was from large to small; node radius was based on its pathway impact value, from small to large; impact value was from small to large. The P-values of pathway enrichment analysis and pathway impact values of pathway topology analysis showed all the matched pathways.


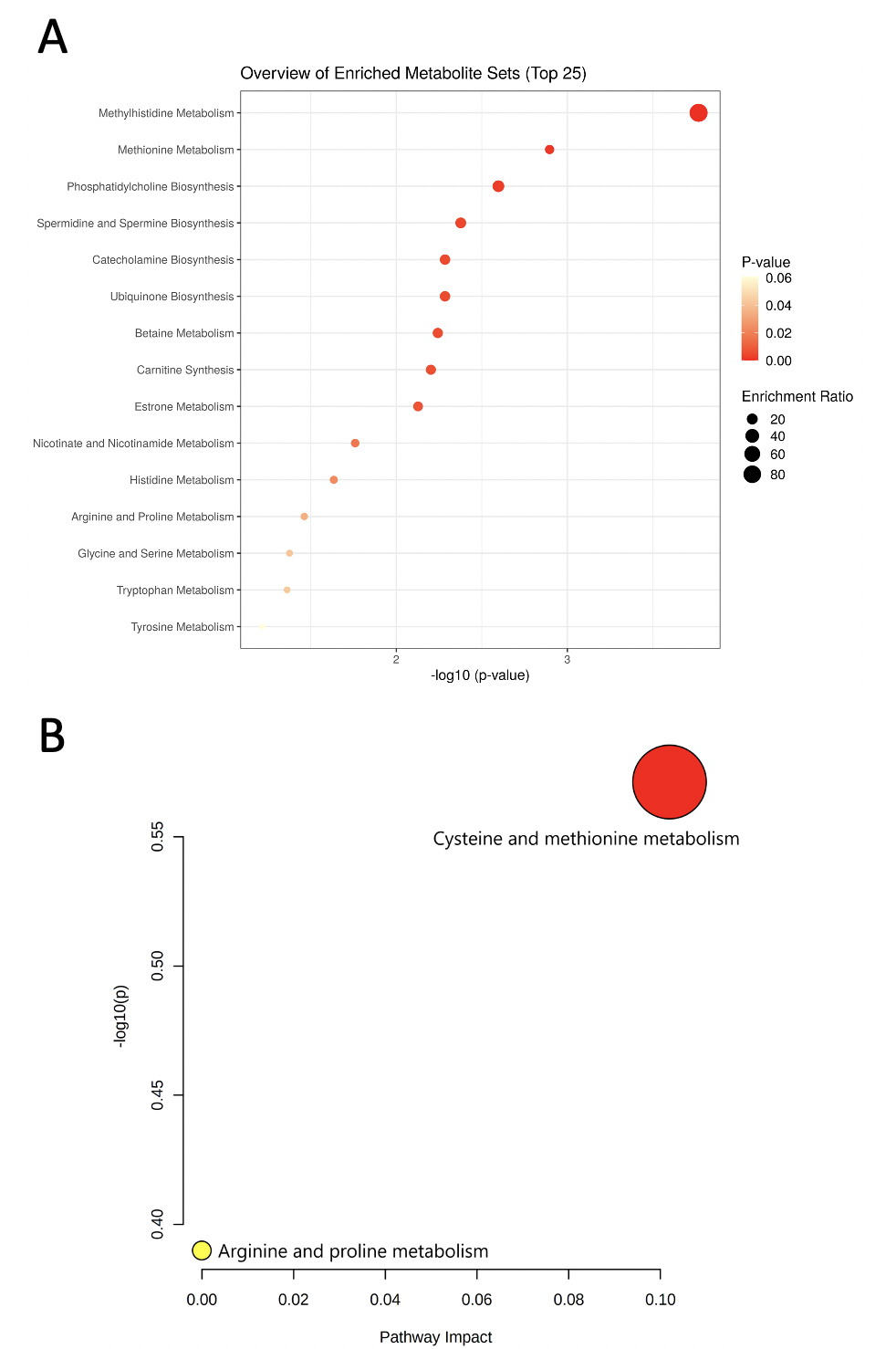


**Fig. S6** Pathway enrichment of down-regulated metabolites between 99.7% O_2_+0.3%CO_2_ and 100%O_2_. (A) The overview of enriched metabolite set. (B) Impact of enriched pathways. The Y-axis was based on the P-value (derived from pathway enrichment analysis), and the X-axis was based on the pathway impact value (derived from pathway topology analysis). Node color was based on P-value, from light to dark; P-value was from large to small; node radius was based on its pathway impact value, from small to large; impact value was from small to large. The P-values of pathway enrichment analysis and pathway impact values of pathway topology analysis showed all the matched pathways.
